# File audit to assess sustained fidelity to a recovery and wellbeing oriented mental health service model: an Australian case study

**DOI:** 10.1186/s13690-019-0377-6

**Published:** 2019-11-23

**Authors:** Cara L. Jones, Frank P. Deane, Keren Wolstencroft, Adam Zimmermann

**Affiliations:** 1 Clinical Psychologist, School of Psychology, University of Wollongong, Wollongong, Australia; 2 Illawarra Institute for Mental Health, Building 22, School of Psychology, University of Wollongong, Wollongong, NSW 2522 Australia; 3 Neami National, Wollongong, NSW Australia; 4 KPMG Australia Services Pty Ltd, Sydney, Australia

**Keywords:** Collaborative recovery model, Evidence-based practice, Implementation, Sustainability

## Abstract

**Background:**

Over the past decade there has been increasing attention to implementing recovery-oriented approaches within mental health service practice and enhancing fidelity to such approaches. However, as is often the case with evidence-based practices, less attention has been paid to the sustainability of recovery-oriented approaches over time. This study sought to investigate whether fidelity to a recovery-oriented practice framework – the Collaborative Recovery Model could be sustained over time.

**Method:**

The study setting was an Australian community managed mental health organisation. A file audit of consumer support plans was undertaken using the Goal and Action Plan Instrument for Quality audit tool (GAP-IQ). The audit tool assessed 17 areas for quality. Consumers (*n* = 116) from a large community managed mental health organisation participated in the study. Sustained fidelity to the Collaborative Recovery Model (CRM) was determined by comparing results from the file audit to a similar audit conducted 3 years earlier.

**Results:**

The file audit revealed a significant increase in fidelity to CRM practices between 2011 and 2014. Fidelity to individual audit items that comprise the GAP-IQ was also found to significantly increase across 16 of the 17 GAP-IQ audit items, with the exception of the ‘Action Plan Review’ audit item.

**Conclusions:**

A comparison of file audit data across different time points within the same setting can provide useful feedback about whether or not a practice is being sustained over time. Although fidelity increased overtime the study design does not allow conclusions that training and coaching practices implemented by the organisation were responsible.

## Background

### Recovery-oriented practice: the collaborative recovery model (CRM)

The promotion of recovery-oriented approaches and their provision in mental health services has increased during the past decade for consumers experiencing and living with mental illness [1]. Recovery-oriented approaches involve fostering personal growth, encouraging active participation in one’s broader community, and empowering consumers to live a valued and meaningful life [2, 3]. Consumers have identified that desired outcomes are not limited to symptom reduction or “getting better” [1], but are more about learning how to live a personally fulfilling life even if symptoms continue to persist [3]. However, challenges are routinely experienced by services in the mental health sector when they attempt to implement recovery-oriented approaches within service practice [4, 5]. Evidence suggests that barriers are influenced by the extent of orientation at the organisational systems and procedural levels [4, 5].

Internationally governments have encouraged or compelled service providers to operate in recovery enhancing ways by linking provision of funding for community-based organisation to this objective [6, 7]. In Australia, the strength of commitment to recovery-oriented practice is easily recognisable in health policy documents [8]. Several Australian National Mental Health Plans have recommended that a recovery orientation should drive service delivery (e.g., Australian Health Ministers, 2009). One of the five priority areas includes addressing “Social Inclusion and Recovery”, and stipulates that mental health providers develop cultures that are founded on and reflective of a recovery orientation [9]. To assist translation at the service and practice level a National Framework for recovery-oriented services was published in 2013 to provide guidance for all people working in the mental health sector for what a recovery-orientation is and how it can be implemented [10]. However, high-level policies and guidance documents are important but not sufficient to ensure recovery-oriented care is delivered. Even when these recovery values are espoused at the level of individual organisations these values may not be translated into practice.

The Collaborative Recovery Model (CRM) is a well-established recovery-oriented approach to working with consumers experiencing a severe mental illness (SMI) [11, 12]. It employs the use of a coaching-style relationship that encourages individual growth, hope and supports people to, “move beyond illness towards one’s best possible self” [13]. The CRM empowers consumers to clarify their values, establish goals and complete action plans that provide them with the direction needed to pursue their vision of a valued life. Progress is reviewed collaboratively between the consumer and their mental health practitioner throughout the course of support. Any barriers to completing an action plan or achieving set goals are identified and modified accordingly [11].

Past studies examining the efficacy of the CRM approach for consumers experiencing a SMI have identified a range of positive outcomes. The approach has been found to be positively regarded by both consumers and mental health practitioners [13, 14] and perceived as being implemented at a high level [14]. Consumers receiving services from CRM trained workers identify components of the approach being delivered (e.g., action planning) at a higher frequency than consumers whose support workers have not been trained in the model [13]. Workers trained in CRM report significantly more positive recovery-oriented attitudes following training [15]. The action planning (therapeutic homework) components of CRM have been associated with more positive service user outcomes [14, 16]. A number of mental health organisations in Australia have adopted the CRM as their primary service delivery model. However, as is common with the implementation of evidence-based practices (EBP’s) in general, barriers have been encountered when implementing the CRM [17].

Studies examining the key factors that facilitate and hinder the implementation of EBP’s have consistently found that staff training is crucial to the successful implementation of EBP [15, 18]. Providing staff with training in the CRM has not only been found to facilitate the implementation of this approach in mental health settings, but also increases the skill and overall confidence of workers in their engagement with consumers [15]. However, staff training in isolation has not resulted in mental health practitioners sustaining their use of recovery-oriented approaches over time [17]. Recent studies indicate that the likelihood of staff transferring newly acquired therapeutic skills, and continuing to use these skills over time, is improved when it is supplemented with regular coaching [11]. Specifically, it has been found that providing staff with training in the CRM that is supplemented by regular supervision or coaching: (i) promotes the transfer of newly acquired skills into everyday clinical practice with consumers, and (ii) is associated with increases in the quality of documented CRM care planning over time [11]. Therefore, the provision of coaching for staff not only has important implications for the successful implementation of CRM practices in mental health settings, but also for the sustainability of these practices over time.

### Sustainability to evidence-based practice (EBP) in mental health settings

While the implementation of EBP in mental health settings has been examined extensively, there has been less of a focus on what happens beyond that point [19]. Sustainability as it relates to EBP is defined as the successful continuation of practice beyond the implementation phase [20, 21]. More recently, the need to examine the sustainability of programs past the implementation stage has emerged as an important research priority [21].

Inadequate funding, staff turnover, and staff resistance to delivering a new treatment approach are factors that have been identified as hindering the sustainability of EBP’s [20]. Facilitators that increase the likelihood that an EBP will be sustained include: staff training that is supplemented with additional training over time, organisations prioritising routine utilisation of an EBP, and funding [20, 22].

### Assessing Fidelity to EBP in mental health settings

Fidelity to EBP refers to how closely services adhere to delivering a particular treatment approach when working with consumers [23, 24]. Assessing fidelity is valuable for organisations as it serves as a quality improvement mechanism for enhancing service provision over time [25]. Research examining fidelity to evidence-based approaches in mental health settings has relied heavily on staff self-reports. Few studies have examined fidelity to EBP by conducting consumer file audits. File audits are advantageous as they provide an alternative and arguably more objective assessment of fidelity [26]. Fidelity to the CRM following staff training has been previously assessed using the Goal and Action Plan Instrument for Quality (GAP-IQ) audit tool [11]. The GAP-IQ measures the quality of CRM care planning within the domains of vision, goal setting, motivational enhancement, action planning and review, as documented in consumer files [11, 13].

### The current study

This paper presents file audit findings from a larger program of evaluation aimed at exploring implementation fidelity to the Collaborative Recovery Model within an Australian community managed mental health service organisation. The organisation is one of the many community managed organisations (also referred to as non-government organisations or NGO’s) in Australia that provide support services to people living with and/or experiencing mental illness and distress. In 2009, the organisation adopted the CRM as a ‘whole of service’ framework to guide service policy and practice. There was high-level leadership support (CEO and Board) for implementation of the program along with the establishment of a mandatory learning and development program to embed the approach across all areas of service practice. All service delivery staff receive 3 days of initial training, a 6 month booster and ongoing annual boosters. Accompanying formal training, staff undertake ongoing parallel process coaching to enhance practice skills, maximise confidence and increase fidelity to the approach.

The broad program evaluation involved three components: 1) a cross-sectional survey with consumer and staff participants assessing perceptions regarding the *importance* of the model’s key practice elements for assisting recovery and the *frequency* by which these are applied, 2) a file audit of consumer support plans, and, 3) interviews with staff across the organisation to assess implementation at the policy and process level. Findings from the cross-sectional survey have been presented in an earlier article [14] and the focus of the current study is on the results of the file audit review. Few studies have examined fidelity to a recovery-oriented model of mental health service provision over time. The aim of the current study is to address this research gap and assess whether fidelity to the CRM was sustained over time following its implementation in the study settings. The results provide an example of how audit review methods can be useful to assess implementation fidelity and sustainability over time.

## Method

### Design

The study involved an audit review of consumers’ files. The results of the file audit from the current 2014 study were compared to an identical file audit that was conducted 3 years earlier in 2011 at the same community managed mental health service. Details regarding file selection procedures are provided below.

### Participants

Participants of the current study were active consumers (*n* = 116) of an Australian community managed mental health service. ‘Active consumer’ refers to people who are currently accessing support as opposed to those on a waiting list. At the time of this study, the service provided support to over 3000 consumers from 29 service site locations across 5 states of Australia.

Recruitment involved a proportional random selection process to select 12 sites from the 29. This resulted in recruitment activities that were distributed across the 5 states as follows: Western Australia (1), Queensland (1), South Australia, (2), New South Wales (4) and Victoria (4). Secondly, lists of active consumers from the 12 sites were generated and a randomisation procedure applied to invite participants. Participation in the file audit review involved consent for researchers to access and review participant files.

Five researchers with a lived experience of recovery (consumer researchers) undertook recruitment and data collection activities. Researchers started at the top of the randomised lists and contacted potential participants initially by phone to inform them about the study and invite participation. To optimise participant representativeness exclusion criteria was limited to age parameters (18-65) and a person’s hospital status and perceived capacity to participate. That is, if a person was at the time of the study an inpatient in hospital and it was deemed by treating staff that they were to unwell to participate they were omitted from the list.

For those participants who were approached to participate, researchers introduced themselves as having a lived experience of mental illness and recovery and provided preliminary verbal information about the study, their role and what participation would involve.

For potential participants who indicated interest in knowing more, a meeting time and place was set with the researcher to discuss the project more fully. Participants were provided with full information (including in written form), invited to discuss and ask questions, and offered time to consider and talk to significant others (e.g. family) about participating. In all communications the voluntary nature of participation was clearly communicated along with options for withdrawing consent. Participants were informed of the protocols to protect participant confidentiality including the use of participant ID numbers on all paper based and electronic data with only researcher access to the ID key. All paper-based data stored in locked filing cabinets with only researcher access and signed consent forms stored separately to paper-based data responses.

Researchers stopped recruitment once participation numbers reached up to 10 per site. Altogether, 263 participants were invited to participate and 117 (44%) agreed to participate after being taken through the informed consent process. One participant subsequently withdrew due to a change in circumstances.

### Procedure

To assess fidelity to the implementation of key practices that form the therapeutic structure of the CRM, the files of the 116 participants were audited. Prior to commencing the file audit, two consumer researchers were trained in how to use the audit tool and then independently audited five files and discussed their ratings to increase consistency. Following this, each researcher audited 58 files each across the twelve selected sites. The two consumer researchers then independently rated *n* = 12 of the other’s files (blind to others ratings) in order to assess interrater reliability. The audit results were then compared to data obtained in a previous study undertaken in several mental health organisations in 2011 [11], however only data from the same organisation was extracted for the purposes of direct comparison in the present study.

### Measures

Fidelity to the CRM in the current study was examined using the *Goal and Action Plan Instrument for Quality* (GAP-IQ) audit tool. This measure was utilised in a similar study that also examined fidelity to the CRM [11] and is provided as an Additional file 1.

### Data analysis

A series of independent sample *t* tests (Bonferroni adjusted *p* < .003) were conducted to determine whether fidelity to the implementation of practices that form the therapeutic structure of the CRM had increased, decreased or remained the same between 2011 and 2014. To verify these effects and assess rates of “high fidelity” a series of Chi Square test of contingencies were also conducted (*p* < .001). This analysis allowed us to determine whether the proportion of “Yes” responses for individual GAP-IQ audit items were significantly different between 2011 and 2014 (see Table 1). We were particularly interested in the proportion of “Yes” ratings since this indicates the highest level of fidelity.

**Table 1:** Mean ratings, Standard Deviations and ‘Yes’ percentages for single item GAP-IQ Fidelity ratings

GAP-IQ Assessment	2011 *N* = 146			2014 *N* = 116		
	*% Yes*	*M*	SD	*% Yes*	*M*	*SD*
1. Overall recovery vision	11.0	0.51	.69	73.3**	1.65	.64**
2. Collaboration between consumer and worker	79.5	1.60	.82	95.7**	1.91	.41**
3. Goal description	30.8	1.18	.64	77.6**	1.66	.67**
4. Goal Importance	21.9	.48	.83	46.6**	.97	.99**
5. Goal Confidence	4.1	.21	.50	85.3**	1.72	.68**
6. Timeframe for goals	3.4	.31	.54	11.2**	.28	.66
7. Level of goal attainment	13.0	.41	.71	54.3**	1.36	.77**
8. Identified barriers/ solutions	12.3	.70	.68	59.5**	1.34	.86**
9. Social support	6.2	.72	.58	55.2**	1.32	.83**
10. Monitoring	16.6	.59	.76	42.2**	1.16	.82**
11. Action plans for goals	31.7	1.10	.73	56.0**	1.38	.78**
12. Action description	39.7	1.17	.78	68.1**	1.51	.78**
13. Action *how* often specified	26.0	.78	.84	69.0**	1.43	.88**
14. Action *when* specified	21.2	.67	.81	50.9**	1.21	.88**
15. Action *where* specified	24.0	.77	.81	69.8**	1.42	.90**
16. Action *confidence* rating	32.2	.68	.93	66.4**	1.39	.89**
17. Action plan *review*	19.2	.53	.80	25.0	.60	.87
Total score		7.50	12.39		22.32	9.92^a^

Note: Excluding the Total Score, Mean scores closer to 2 reflect greater fidelity to the CRM

^a^*t*(60) = − 10.39, ** *p* < .001. ** Chi-square significant at *p* < .001 level

## Results

Of the 116 participants, 55% were male and 45% were female. The mean age of participants was 42.9 years (*SD* = 11 years; range 20 to 69 years). Participants were identified as holding primary diagnosis categories of; schizophrenia (41%), depression (20%), bipolar disorder (14.3%), schizo-affective disorder (11.4%), anxiety (2.9%), and other (10.7%). Length of time receiving support from the service was; less than 1 year (10%), 1–2 years (46%), 2–3 years (26%), 3–4 years (10%), 5+ years (7%). Fourteen percent of participants were of Non-English speaking background and 6% identified as Aboriginal or Torres Strait Islander. Participants as a group had a total of 62 different ‘key workers’ who had primary responsibility for the coordination and delivery of their mental health support.

Interrater reliability on the subset of 12 independently rated files was calculated using Kappa. Coefficients ranged from a low of .39 to 1.00 with a median of .85. Using the descriptors provided by [27], 1 of the 17 codes had “Fair” interrater reliability (0.21–0.40), 3 codes were “Moderate” (0.41–0.60), 3 codes were “Substantial” (0.61–0.80) and the remaining 10 codes had “Almost perfect agreement” (0.81–0.99).

An independent sample *t*-test of the total GAP-IQ score revealed a significant increase in the overall fidelity to the CRM between 2011 and 2014. Table 1 outlines the mean and standard deviation ratings across 2011 and 2014 for the 17 GAP-IQ CRM items. A series of independent samples *t*-tests were conducted for individual items using a Bonferroni adjusted *p*-value of .003.

Table 1 below outlines the mean ratings, standard deviations and ‘Yes’ percentages for single item GAP-IQ fidelity ratings.

With the exception of the ‘Action Plan Review’ item on the GAP-IQ audit tool, the results from a series of Chi Square contingencies were all significant indicating a significantly higher proportion of “Yes” ratings in 2014 than in 2011 for 16 of the 17 items (all *p* < .001). The percentage “yes” responses in 2011 revealed four items that were particularly low (Time frame for goals, 3.4% to Recovery vision, 11%). All showed improvements by 2014 but, the provision of “Timeframe for goals” remained low (11.4%).

### Variation between service sites

Since there were only an average of 10 files selected from each service site only descriptive data is provided to portray the variation in GAP-IQ ratings. There was considerable variability between the 12 sites with the best performing site having a mean of 31.00 (SD = 2.08) on the GAP-IQ and the poorest performing site having a mean of 17.10 (SD = 7.55). When sites were categorised into High (31–27), Medium (26-22) or Low (21-17) groupings based on mean scores, there was only one site in the High group, with 6 in the Medium group and 5 in the Low group.

## Discussion

The aim of this study was to determine whether fidelity to CRM practices had remained the same, increased or decreased between 2011 and 2014. Overall, an increase in fidelity to CRM practices was observed for 16 of 17 GAP-IQ items, with the exception of Action Plan Review item. Fidelity to Action Plan Review did increase between 2011 and 2014 (19.2 to 25%) but this increase was not significant and reflected poor fidelity to this component of the CRM. In addition, there were several components that had particularly low fidelity ratings in 2011 but all had subsequently improved in 2014 (e.g., Recovery vision, Time frame for goals, Goal confidence, Social support). It is possible that feedback from the 2011 audit to trainers within the organisation contributed to greater emphasis on these components of the CRM model in training activities. However, specification of time frames for goals remained low in 2014. It is possible that this aspect of the goal planning process was infrequently specified since there is no specific prompt on care planning forms to specify the time frame. Thus, revision of the goal plan forms to include a specific time frame prompt along with a focus during training activities is warranted.

Although there were some components of CRM practice that could clearly be improved, overall fidelity to CRM practices increased over time in the current study and was on average high in this organisation. However, currently no benchmarks exists for the fidelity of organisations to CRM practices. Without a benchmark, it is unclear whether fidelity ratings in the current study are acceptable. To determine whether fidelity ratings in the current study were practically meaningful, we compared the study results to a similar study which examined fidelity to therapeutic homework implementation and completion within a CRM framework based on 122 case manager self-reports [28]. Fifty-eight percent of case managers reported that they specified the frequency of homework practice “often” or “almost always” and 49% similarly specified the location, with 84% indicating they reviewed the homework at the beginning of the next session [28]. When compared to equivalent components of the 2014 audit, ratings of how often (frequency specified) an action plan was completed was higher (69%), where (location) an action should be completed was higher (70%) but the frequency of evidence that homework has been reviewed was lower (25%). Caution is needed in comparing these rates of action plan components due to the difference in methods used between the studies (self-report versus file audit review). The difference in rates that mental health workers indicate they review the homework is likely due to methodological differences where self-report has clearly produced higher rates than in the current study where documented evidence of the review process was required.

Although there may be no external organisations that currently have data to support benchmarking of CRM, there is the potential to use GAP-IQ scores to benchmark between different service units within the organisation. There was variability between sites on overall GAP-IQ mean scores. Although the number of files available for each site did not allow statistical analysis of this variability, descriptively it was clear that some sites performed substantially better than others with one site in particular a stand out. Such site-specific data has the potential to be used to guide service level training in order to be able to improve overall adherence to treatment and quality protocols.

To our knowledge, the current study one of the first to examine sustained fidelity to recovery oriented practices over time. One prior study [20] that examined sustainability of evidence-based practices (EBP) in community mental health agencies included a recovery-oriented EBP. This EBP was Illness Management and Recovery [29] and for the 12 agencies who implemented this approach 8 (66.7%) indicated they had sustained it 2 years later and 3 (25%) at 6 year follow-up [20]. Further, the IMR was described as the EBP that had the most significant adaptations out of the five EBPs reviewed. Judgements of sustained practices were based on interviews with site and state agency leaders. Thus, the differences in methods for assessing sustained practices and varying follow-up time frames make comparisons between studies problematic. In addition, although IMR might have been judged by site leaders to be continuing, the adaptations described raise questions about the fidelity to the original IMR program.

Given different methodology between the studies it is difficult to determine whether discrepancies in fidelity to particular components are a function of self-report versus file audit methods of data collection. Where appropriate to we would encourage more studies to utilise independent rater audits of files using the GAP-IQ or relevant fidelity tools even if these are via interviews with key informants. Such methods are likely to reduce potential bias (e.g., social desirability) that may inadvertently influence perceptions of current practice.

Irrespective of the availability of benchmarking reference points, repeat measures of file audit data provide important feedback about areas of focus for improving fidelity in the future. Overall, an acceptable level of fidelity to the action planning components of CRM practices was broadly suggested by the results in the current study, however, review of action plans needs improvement. If action plans are not consistently reviewed this can potentially be demotivating for consumers and diminish the perceived importance of the planned therapeutic homework activities. In ongoing coaching and booster training highlighting the importance and skills associated with reviewing progress on prior action plans could be emphasised. This might involve clarifying the importance of these reviews to provide; encouragement and reinforcement for consumer effort and work done, clear connections for the links between action plans, goal attainment and valued life directions, and revising and planning future therapeutic homework activities. Repeat file audits will provide the opportunity to determine whether changes in coaching and training result in improvements in particular domains.

### Strengths and limitations

In the current study, is it is not possible to make causal statements about the effects of coaching and training effects on fidelity and sustainability of CRM in this organisation. Although the same audit tool (GAP-IQ) was used at both time points, it is possible that different file selection processes and different audit raters may also have affected the results. However, the use of the same randomisation process used in the current study could be replicated in future studies in order to reduce these potential sources of variability.

The response rate (44%) for participation in this study was relatively low and may influence the reliability of the findings presented. However, the response rates may also be the result of steps taken in the study design to limit bias and potential perceptions of coercion. For example, recruitment activities were undertaken by consumer researchers who had no prior relationship with participants. Recruitment rates may have been higher if consumers had been recruited by staff they already knew. Moreover, participation in mental health research is often influenced by clinician decision making about whether a person is suitable or likely to want to be involved [30]. To improve opt-in, opt-out choice the study limited exclusion criteria to that of age and current hospital usage. Given that participation in this study was a component of a larger evaluation process that included surveys, it is possible that consumers decided not to participate because of other elements also being requested.

## Conclusion

The current study is noteworthy as it is one of the first studies to examine fidelity to a recovery-oriented model of mental health service provision and sustainability over time. Providing clinicians with training and ongoing coaching in evidence-based practices (EBP’s) is thought to not only increase fidelity to EBP’s within organisations, but also the frequency that clinicians will deliver, and consumers will receive the specific components of an EBP. While the extent that recovery oriented approaches can be sustained over time in mental health settings is not known, results from the current study suggest that the community managed mental health service successfully: (i) sustained their use of CRM practices over time, and (ii) increased their fidelity to CRM practices across a 3-year period. It remains for future research to determine whether these sustained and relatively high fidelity practices are associated with staff coaching, booster training and/or high-level organisational support.

## Supplementary information

**Additional file 1.** Gap IQ.

## Data Availability

The datasets generated and/or analysed during the current study are not publicly available to comply with University of Wollongong Ethics approvals but are available from the corresponding author on reasonable request and HREC application. The measure used in this study is included in the Additional file 1.

## Abbreviations

CRM: Collaborative Recovery Model
EBP: Evidence-based practice
Gap IQ: Goal and Action Plan Instrument for Quality
SMI: Serious mental illness
