# Supplementary material for: File audit to assess sustained fidelity to a recovery and wellbeing oriented mental health service model: an Australian case study
Source: Arch Public Health. 2019 Nov 23;77:50. doi: 10.1186/s13690-019-0377-6 (PMC6874813; doi:10.1186/s13690-019-0377-6)
Supplement: Supplementary file 1 — Additional file 1. Gap IQ. [file 13690_2019_377_MOESM1_ESM.pdf]

Client file audit protocol

Client File Audit – Care Plan Quality

Please ensure that the care plan being audited for quality is the MOST RECENT care plan in the audit period for this client

Date of audit __________ Audit completed by ________________

Consumer ID # ___________

Date this client started receiving services from this organisation ___________________

		0 = No evidence of implementation<br>1= protocol available
1	Camera completed	
2	Compass completed	
3	Map completed	

Goal and Action Plan Quality Audit Instrument

1. Is there an overall recovery vision	No	No written record that meaning, hopes, dreams, values and/or preferred identity that the person wishes to head towards or practice including in his/her life were discussed with the consumer.
	Partial	Written record that hopes, dreams and values for the future has been discussed, but the goals selected do not appears to be in line with the consumer's values or there is no record that the consumer has been asked "why" they would like to achieve their set goals.
	Yes	Written record that hopes, dreams and values for the future has been discussed. There is a direct link between meaning, hopes and dreams the individual holds for their future and goals selected within case-management and these are documented (e.g. " Consumer reported that getting his own shopping (goal) would lead him to feel more independent (recovery vision)).
2. Collaboration between consumer and worker	No	Language in the care plan does not suggest that collaboration between consumer and worker occurred when identifying care plan goals (e.g. "consumer was instructed to work on medication adherence", "consumer was provided with goals set out by his mental health team").Or there is language in the file that describes the consumer or their goals in negative terms (e.g. insight less, unrealistic, unmotivated).
	Yes	Language in the care plan indicated that collaboration between worker and consumer occurred when developing goals. Goals are recorded in layperson's terms void of technical jargon.
3. Goals	No	No case-management goals are recorded.
	Partial	Some goals are recorded– yet they are not clearly defined making measurement difficult (e.g. to feel better, to be happier)

	Yes	Goals are recorded and defined so that a clear outcome is measurable (e.g. to do my own shopping, improve my medication taking, to find a job).
4. Goal Importance	No	No record that the consumer's perceived importance of goals selected or prioritisation of the care plan goals.
	Partial	A written record that the consumer's perceived importance for each goal has been considered and resources allocated accordingly (e.g. consumer stated that ___ goal was most important for them, so the session was spent working toward this").
	Yes	A record that goal importance has been ranked numerically or ordered and resources allocated accordingly (e.g. Consumer placed goals in order of importance (1, 2, 3) so session time and tasks were allocated with this in mind).
5. Confidence	No	No written record that consumer's level of confidence was rated for the goals selected
	Partial	Written record that confidence was asked (e.g a statement or rating) in relation to one of the goals but not others.<br>A written record that consumer confidence was assessed, yet goals were not adjusted to enhance the consumers self efficacy related to goal attainment
	Yes	A written record that confidence was asked in relation to each case-management goal and goals were adjusted to enhance the consumer's confidence for goals attainment.
6. Time frame for Goals	No	No time frame established for goals.
	Partial	Some record of a time frame for goal completion, but this is vague (e.g. end of the year, rather than a specific date). Or the timeframe seems unrealistic for the type of goal selected? (e.g. to commence and complete a TAFE course within 3 months).
	Yes	Written record of an established time frame and a date set for the review period.
7. Levels of goal attainment	No	No varying levels of goal attainment defined for the treatment goals recorded.
	Partial	Some but not all of the case-management goals have different levels of goal attainment defined and recorded.<br>Levels for goals are defined, yet they are not behaviourally defined making outcome difficult to measure. (E.g. lacks specifications such as; frequency, what, where and with whom).
	Yes	Levels for each of the case-management goals are specified and are behaviourally defined (e.g. frequency, what, where, with whom) so outcome can be clearly measured.
8. Identifying and problem solving barriers to goal attainment (coping planning)	No	No written record that barriers to goal attainment are identified in the care plan. OR if no barriers are described, there is also no evidence that potential barriers were discussed and solutions to address these identified.
	Partial	A written record that some potential barriers were discussed, however no problem solving around these is evident. (E.g. lack of money may be problems yet attempts to assist budgeting or identify alternative solutions are not evident).<br>Only some of the treatment goals were recorded as being the focus of coping planning.

	Yes	A written record that barriers and potential solutions for each of the treatment goals have been discussed.
9. Social Support	No	No written record that social support was enlisted to assist with goal attainment.
	Partial	Written record that some social support was identified - either only at a service level (case- manager) or personal level (family member).
	Yes	Written record that social support was identified to assist with goal attainment, both at a personal and service level. Roles for different members have been discussed and outlined. This can include practical (e.g. transportation), emotional (e.g. to hear the other persons concerns) or informational support (e.g. Information on harm minimisation or side effects of medication etc.)
10. Monitoring	No	No written record regarding how goal progress will be monitored.
	Partial	General written reference made to monitoring progress (e.g. will check progress with consumer).
	Yes	Specific written record of how progress of behaviours in specific settings will be monitored (e.g. In addition to homework tasks, consumer has agreed to keep a graph of his number of walks at the oval or mood diary)
11. Action Plans for goals (General rating)	No	No record that discussions about pathways or strategies for any of the goals has taken place (e.g. steps to the goals),
	Partial	A written record that some of the case-management goals have plans developed outlining how the goal will be achieved. Or a written record that the treatment goals have plans developed, yet these are not defined or specified clearly.
	Yes	A written record that all goals selected have clear pathways of how to attain the goal and the specific details about when, where and how the goal will be carried out. Target goal must be specified in action plan.
12. Action **Description**	No	Not completed
	Partial	Item attempted but insufficient or inappropriate information
	Yes	Item completed well. A description should be provided regarding what the actual homework assignment is. The task should be described in sufficient detail that the client and clinician have a clear understanding of what the task is.
13. Action **How often** specified	No	Not completed or inappropriate (e.g., run in the park).
	Partial	Item attempted but insufficient or inappropriate information. Description is not specific, but an attempt has still be made to record a response. e.g., “As required”, “when I feel like it”.
	Yes	Item completed well. Clearly describes the number of times the task should be completed (e.g, numeric 2x, Each morning, Daily)
14. Action **When** specified	No	Not completed or inappropriate response (e.g., run in the park)
	Partial	Item attempted but insufficient or inappropriate information. Description is not specific, but an attempt has still be made to record a response. (e.g., When required, when I feel in the mood, when I think about it)
	Yes	Item completed well. Clearly describes the time and/or date that the specific task is to be completed. (e.g., Morning, afternoon, night; 12pm, 3am etc; Monday, Tuesday etc.; Each morning before breakfast)

15. Action **Where** specified	No	Not completed or clearly inappropriate (e.g. run every morning). Where the task is to be done should be completed.
	Partial	Item attempted but insufficient. Description is not specific (e.g. Wherever I get a chance)
	Yes	Item completed well. Clearly describes where the task should be completed. Specific location. (e.g., Home, around the block; at the hospital)
16. Action **Confidence Rating** provided	No	Not completed. No number on the confidence scale is circled.
	Partial	The confidence scale has a number circled that is less than 70
	Yes	The confidence scale has a number from greater than or equal to 70 circled.
17. Action plan **Review**	No	Not completed. Neither a rating nor comment is provided to indicate a review was conducted.
	Partial	There was either a comment made OR a formal rating, but not both.
	Yes	Item completed well. There is a formal rating of the quantity or quality made by either the client or mental health worker. There is a comment made indicating a review was conducted.
